# Supplementary material for: Causal Relationship between Aging and Anorexia Nervosa: A White-Matter-Microstructure-Mediated Mendelian Randomization Analysis
Source: Biomedicines. 2024 Aug 16;12(8):1874. doi: 10.3390/biomedicines12081874 (PMC11351342; doi:10.3390/biomedicines12081874)
Supplement: Supplementary file 1 [file biomedicines-12-01874-s001.zip › Supplementary Figure/Figure S8/Leaveoneout plot of MR analysis for TL to ubm-b-1892.pdf]

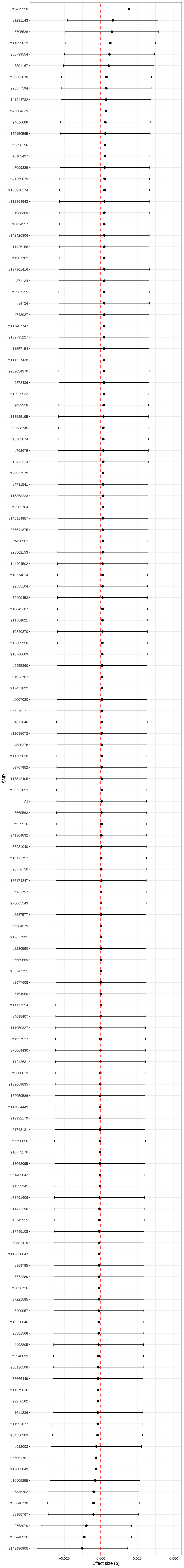

rs9419958
rs1291143
rs7705526
rs11699829
rs56799554
rs3891167
rs28363070
rs28577594
rs141214782
rs45604339
rs4616688
rs150150565
rs9398196
rs8102497
rs7099229
rs41269079
rs188918174
rs112394943
rs1985369
rs6054257
rs142426306
rs11426156
rs1907702
rs137901416
rs871134
rs2967355
rs4724
rs4743037
rs117407747
rs139795227
rs11557154
rs111527438
rs202034370
rs9878436
rs12925933
rs429358
rs113525195
rs2538745
rs3785074
rs762679
rs12412214
rs79977579
rs4731541
rs116863223
rs2282764
rs145114957
rs376641875
rs450962
rs28502153
rs144204502
rs10774624
rs2555104
rs59409453
rs10845387
rs11584821
rs10840270
rs12369950
rs10768683
rs9955360
rs1023767
rs12451892
rs6007020
rs76219171
rs611646
rs11085072
rs4530278
rs11769630
rs3767952
rs117512405
rs66731853
All
rs6669563
rs939916
rs41304832
rs77231040
rs10112752
rs6776756
rs185174247
rs131797
rs76065543
rs6587577
rs6584579
rs17677991
rs2230590
rs6659669
rs55747751
rs2977608
rs7164950
rs11117354
rs4695407
rs111950327
rs1957937
rs75664430
rs11212631
rs9600019
rs139669835
rs182059586
rs117034449
rs12932179
rs61748181
rs7790856
rs10773176
rs13062095
rs61405042
rs1332941
rs78491606
rs11412296
rs5742915
rs17445108
rs73581419
rs117630647
rs869785
rs7772289
rs2056726
rs7221585
rs7209057
rs13230646
rs6881568
rs4498805
rs9940099
rs80116508
rs76666449
rs11579626
rs2276182
rs1611236
rs11991877
rs34550383
rs932002
rs56061761
rs17803849
rs10905255
rs6536702
rs35640778
rs8105767
rs2763979
rs35446936
rs143190905
SNP
-0.025
0.000
0.025
0.050
Effect size (b)
